# Advertising for Demand Creation for Voluntary Medical Male Circumcision

**DOI:** 10.1097/QAI.0000000000001039

**Published:** 2016-10-06

**Authors:** Nicholas Wilson, Sasha Frade, Dino Rech, Willa Friedman

**Affiliations:** * Department of Economics, Reed College, Portland, OR;; † The Centre for HIV and AIDS Prevention Studies (CHAPS), Johannesburg, South Africa; and; ‡ Department of Economics, The University of Houston, Houston, TX.

**Keywords:** advertising, conditional cash transfer, epidemiology, HIV, South Africa

## Abstract

To measure the effects of information, a challenge, and a conditional cash transfer on take-up of voluntary medical male circumcision (VMMC).

**Design:**

A randomized, controlled experiment with 4000 postcard recipients in Soweto (Johannesburg), South Africa.

**Methods:**

We examined differences in take-up of several decisions in the VMMC cascade between the control arm and each of several intervention arms using logistic regression.

**Results:**

Logistic regression analysis indicated that the group offered US $10 as compensation and the group challenged with “Are you tough enough?” had significantly higher take-up of the VMMC procedure than did the control group [odds ratios, respectively, 5.30 (CI: 2.20 to 12.76) and 2.70 (CI: 1.05 to 6.91)]. Similarly, the compensation group had significantly higher take-up of the VMMC counseling session than did the control group [odds ratio 3.76 (CI: 1.79 to 7.89)]. The analysis did not reveal significantly different take-up of either the VMMC counseling session or the procedure in the partner preference information group compared with the control group [odds ratios, respectively, 1.23 (CI: 0.51 to 2.97) and 1.67 (CI: 0.61 to 4.62)]. The analysis did not reveal significantly higher take-up of the VMMC nurse hotline in any intervention group compared with the control group [odds ratios for US $10, information, and challenge, respectively, 1.17 (CI: 0.67 to 2.07), 0.69 (CI: 0.36 to 1.32), and 0.60 (0.31 to 1.18)].

**Conclusions:**

Among adult males in Soweto, South Africa, compensation of US $10 provided conditional on completing the VMMC counseling session compared with no compensation offer and a postcard with a challenge, “Are you tough enough?” compared with no challenge, resulted in moderate increases in take-up of circumcision.

## INTRODUCTION

Evidence from randomized controlled trials conducted in Kenya,^1^ Uganda,^2^ and South Africa^3^ indicates that voluntary medical male circumcision (VMMC) reduces HIV acquisition by 51–76%. Based on these findings, the international donor community is aiding scale-up of mass VMMC campaigns in 14 high HIV prevalence, low male circumcision prevalence priority countries in sub-Saharan Africa.^4^ However, take-up of this fully subsidized health service remains low.

Existing literature on demand for VMMC highlights several factors that may be inhibiting VMMC take-up. First, opportunity costs of foregone wages during the recovery period^5–8^ and transportation costs associated with the counseling session/procedure^9^ seem to be large impediments to receiving VMMC. Second, individuals are concerned about pain associated with the procedure and recovery period.^5,6,8,10^ Third, men are concerned that VMMC will reduce sexual performance and pleasure.^5–8,10^ Fourth, the fact that explaining to individuals that VMMC reduces the acquisition of HIV and other sexually transmitted infection (STI)s seems to increase the acceptability of VMMC^5,7,10^ suggests that individuals may be uninformed about possible benefits of VMMC.

To assess the magnitudes and relative importance of these barriers, we conducted a randomized controlled trial. In this study, we randomly assigned postcards offering a conditional cash transfer for completing the VMMC counseling session, a challenge message, and information on a possibly previously unknown benefit of VMMC, through 4000 postcards in Soweto township.

## METHODS

### Study Setting, Design, and Participants

Soweto, our study setting, is a relatively poor township in Johannesburg, South Africa. Gauteng province, where Soweto is located, has the lowest prevalence of male circumcision, 25.2%^11^, and the second highest number of people living with HIV of any province in South Africa.^12^ We conducted our study in collaboration with the Centre for HIV and AIDS Prevention (CHAPS), which currently operates 23 VMMC sites in Gauteng. The Human Subjects Research Committee (Medical) at the University of Witwatersrand, Johannesburg and the Institutional Review Board at Reed College provided human subjects research ethical approval for our study.

Outreach workers distributed postcards individually sealed in envelopes in a blinded, prespecified order to households, following a random walk method. In this random walk, outreach workers began distribution at prespecified locations, leaving the next postcard at every fifth house with an adult present, using coin-flips to determine the path at each intersection. The prespecified order randomized each postcard type and stratified on timing and location of distribution. Importantly, our statistical analysis compares VMMC take-up across groups of postcard recipients rather than with nonrecipients, which reduces many spillover concerns that complicate other studies. If recipients shared their postcards with those who had not gotten any postcard, and this person visited a clinic, this does not compromise the design because the second person was not in another experimental group.

Men who presented a study postcard at a participating CHAPS clinic were asked to provide written informed consent.

### Interventions

All postcards offered basic information about the HIV risk-reduction benefits of circumcision and clinic hours, and an offer of refreshments for male respondents, aged 18 years and older, who participated in a VMMC consultation. Subsets of these were also offered a conditional cash transfer (ie, R100, approximately US $10), information on a possibly previously unknown benefit of VMMC (ie, possible partner preference), or a challenge message (ie, “Are you tough enough?”). Our cross-cutting randomization produced 4 study arms of equal size (ie, 1000 postcards each). Control postcards included basic information that VMMC reduces HIV acquisition by 51–76% and the names and workdays of participating clinics. The offer of light refreshments served as an incentive for the recipient (or another adult male) to bring the postcard with him if he chose to come to a clinic. Requiring individuals to bring the postcard to the clinic for refreshments allowed us to measure take-up of multiple decisions in the VMMC cascade and observe how this varied across study arms.

“Money” postcards offered R100 (ie, approximately US $10) as compensation for attending a VMMC counseling session at a participating clinic in addition to the information on the control postcard. “Information” postcards stated that a recent survey indicated that among partners of uncircumcised men, 2 of 3 would prefer that their partner be circumcised in addition to the statements in the control postcard. “Challenge” postcards added the statement, “Are you tough enough?,” to the control postcards to frame the statement about the 51–76% reduction in HIV acquisition.

All transfers were disbursed conditional on attending a VMMC counseling session at a participating clinic. Distribution of postcards began on June 30, 2014. All postcards stated an offer expiration date of August 29, 2014, meaning each postcard had a redemption period of approximately 2 months.

### Data Collection and Statistical Analyses

We collected data on 3 outcomes related to the VMMC cascade (ie, the series of steps required to complete VMMC). These 3 outcomes were (1) calling or texting the VMMC hotline, (2) completing the counseling session, and (3) completing the procedure. Each type of postcard had a different phone number, which meant that each call could be linked with the type of postcard seen by the caller. For each individual who brought a postcard to a participating clinic, a nurse recorded the postcard information along with whether the individual completed the counseling session and whether he underwent the procedure. We also collected demographic information and past risky sexual behavior from individuals who brought postcards to the clinics. No individuals under age 18 presented postcards.

The primary outcomes of interest were uptake of the counseling session and of the procedure, as well as the initial possible step of calling the VMMC hotline. For each of these 3 outcomes, the independent variable is a binary variable equal to 1 if the participant completed the step and equal to zero if otherwise. Our primary analysis uses logistic regression to compare uptake for each of these outcomes in each of the intervention groups to uptake in the control group. We used Stata Version 13.1 (StataCorp) to conduct our statistical analyses and all statistical tests were two sided with a *P* value of less than 0.05.

The trial specified a sample size of 4000 postcards, divided into 4 study arms of equal size. In our power calculations, we analyzed the sample size required to test whether a single intervention generated a statistically larger take-up. We assumed a two-sided test with an alpha of 0.05 and a goal of 90% power. In addition, we assumed that 5% of control postcard recipients would obtain VMMC and that an intervention postcard (ie, financial compensation of approximately US $10, information on partner preferences, the challenge “Are you tough enough?”) would increase this number by 5 percentage points. The required sample size in each group was 621, or 2484 summed across the 4 different study arms. We chose a sample size of 4000 to ensure that we were able to identify an even smaller intervention effect than 5 percentage points.

## RESULTS

### Characteristics of Respondents

Table 1 displays basic characteristics of respondents who presented study postcards at participating clinics. Of the 4000 postcards we distributed, 74 (1.9%) were returned by men seeking VMMC counseling at a participating CHAPS clinic.

**TABLE 1.**
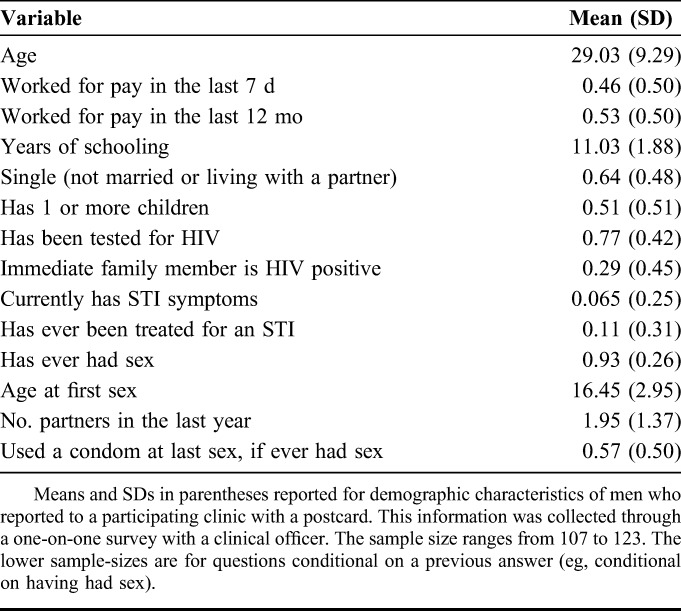
Descriptive Statistics for All Men Completing VMMC Counseling Session

Among respondents who subsequently presented a postcard at a participating clinic, 91% said they had gotten it directly from the distributor and 9% said they had gotten it from a friend or family member. The average age of men who completed the survey was 29 years. Just under one-half of respondents worked for pay in the last week, and exactly one-half did so in the last year. The average year of schooling among the respondents was roughly 11. Approximately one-half of men had at least 1 child and 67% were neither married nor living with their partner. Twenty-six percent of respondents reported that an immediate family member was HIV positive. More than three-quarters of respondents had ever been tested for HIV. Nine percent had ever been treated for any STI, and 6% currently had symptoms. Nearly all respondents reported ever having had sex (90%), with the average age of debut at 16 years. The average number of partners in the last year was nearly 2 and one-half of respondents reported using a condom the last time they had sex.

### Effects on VMMC Hotline

Table 2 presents the effects of the intervention postcards on take-up of several decisions in or associated with the VMMC cascade. Column (1) in Table 2 shows the effects of each of the 3 main interventions on whether a recipient contacted the hotline. For none of the 3 interventions did we observe a statistically significant effect on the likelihood the study participant called or sent a text message to the VMMC hotline (for compensation of US $10, challenge “Are you tough enough?,” and information on partner preference, respectively: odds ratio (OR) 1.18, 95% CI: 0.67 to 2.07; OR 0.60, 95% CI: 0.31 to 1.18; OR 0.69, 95% CI: 0.36 to 1.32).

**TABLE 2.**
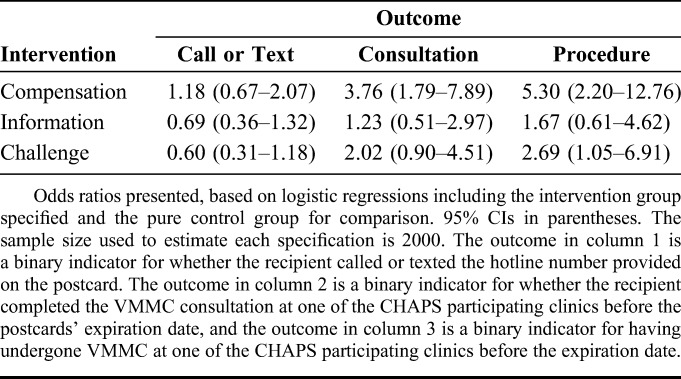
Effects of Compensation, Information, and Challenge on Various Steps in VMMC Cascade

### Effects on VMMC Counseling Session

Column (2) in Table 2 shows the effects of each of the 3 main interventions on take-up of the VMMC counseling session. Uptake of the VMMC counseling session was significantly higher in the US $10 group than that in the control group (OR 3.76, 95% CI: 1.79 to 7.89). Uptake in neither the challenge group nor the “2 of 3 prefer a circumcised partner” group was significantly different than that in the control group (ORs, respectively, 2.02 and 1.23; 95% CIs, respectively, 0.90 to 4.51 and 0.51 to 2.97).

### Effects on VMMC Procedure

Column (3) in Table 2 presents the effects of the intervention postcards on take-up of the VMMC procedure. Take-up of the VMMC procedure was significantly higher in the US $10 group and in the challenge group than that in the control group (ORs, respectively, 5.30 and 2.69; 95% CIs, respectively, 2.20 to 12.76 and 1.05 to 6.91). Take-up of the procedure was not significantly different in the “2 of 3 prefer a circumcised partner” group than that in the control group (OR 1.67, 95% CI: 0.61 to 4.62).

### Cost-Effectiveness

This intervention is extremely low cost. Combined with noticeable impacts on uptake, this implies that the cost-effectiveness is relatively high. Printing and distribution of each postcard cost approximately US $2, and the cash transfer costs US $10 per consultation. Refreshments for each consultation cost approximately US $1. The high conversion rate (110 of the 123 men who came for a consultation stayed for the procedure) and only needing to pay the latter 2 costs for those who chose to use the voucher kept costs low. However, the number of postcards needed to encourage each additional circumcision may be considered high, increasing costs. The expected effect on take-up from the addition of the compensation offer on the postcard relative to the basic postcard was 2.5 percentage points. This gives a cost per additional circumcision of US $91. The fact that Soweto is a high HIV prevalence area implies that 1 VMMC generates approximately 1/15–1/5 of an HIV infection averted.^13^ Thus, we estimate that this intervention costs between US $450 and US $1350 per HIV infection averted, excluding clinical costs.

## DISCUSSION

The results of our randomized controlled trial indicate that offering economic compensation for the VMMC counseling session was effective at increasing take-up of the VMMC procedure. Similarly, including the challenge, “Are you tough enough?,” was also effective at increasing VMMC take-up. In contrast, providing the information that, among partners of uncircumcised men, 2 of 3 would prefer that their partner be circumcised was not effective at increasing VMMC take-up.

At least 2 other studies have conducted randomized controlled trials testing the effect of conditional economic compensation on VMMC take-up. The magnitude of the main effect reported in the first study is very similar to the effect of economic compensation that we report in the current analysis. That is, in Kenya, an offer of US $8.50 in food vouchers disbursed conditional on completing the VMMC procedure increased VMMC uptake relative to the control arm more than 4-fold.^14^ The second study also found similar effects of conditional compensation of approximately US $11 on VMMC uptake in Kenya.^15^

The finding that posing the challenge, “Are you tough enough?,” was effective at increasing VMMC take-up provides scarce experimental evidence for the largely nonexperimental literature citing (concern about) pain^5,6,8,10^ and (expected) diminished sexual performance as major factors limiting demand for VMMC.^5–8,10^ Although many largely nonexperimental studies propose that explaining to individuals that VMMC reduces the acquisition of HIV and other STIs increases the acceptability of VMMC,^5,7,10^ our results indicate that the responsiveness of VMMC take-up to further information campaigns, or at least information campaigns citing partner preference for circumcised men, may be limited.

Focus group discussions among study participants point to a possible explanation for the effectiveness of what was relatively small financial compensation: many of the men reported that they were thinking of taking up the VMMC procedure anyway. The extremely high rate of conversion (ie, take-up of the procedure conditional on coming for the counseling session) also supports this explanation. If this explanation is true, then it suggests that the use of financial compensation in the context of VMMC may not be coercive. Men induced to complete the VMMC procedure may be likely to be considering the VMMC decision seriously already and compensation simply may be a nudge. In this study, payment conditional on the consultation rather than the procedure provided additional assurance against coercion.

Interventions generated VMMC procedures that would not have occurred during redemption period in the absence of the interventions. The ratio of procedures conducted among men who would have not received the procedure in the absence of the US $10 offer to those who would have performed so anyway was approximately 5 to 1. For the challenge intervention, this ratio was slightly less than 3 to 1.

This study has a few limitations. First, respondents were only given between 1 and 2 months to redeem their vouchers. A longer redemption period may have generated higher take-up. Second, because we chose not to conduct a baseline survey of postcard recipients (which could have biased the findings by increasing salience of issues around circumcision), we do not know background characteristics of recipients. Without a baseline survey, we cannot know which postcard recipients were uncircumcised at baseline, and we cannot compare the demographic characteristics of those recipients who did and did not visit a clinic. Although this lack of a baseline may limit the nuances we can uncover, the randomized design removes concerns about omitted variables, reinforcing the credibility of the study findings.
